# Adverse neonatal outcomes in women with pre-eclampsia in Mulago Hospital, Kampala, Uganda: a cross-sectional study

**DOI:** 10.11694/pamj.supp.2014.17.1.3014

**Published:** 2014-01-18

**Authors:** Paul Kiondo, Nazarius Mbona Tumwesigye, Julius Wandabwa, Gakenia Wamuyu-Maina, Gabriel S Bimenya, Pius Okong

**Affiliations:** 1 Makerere University College of Health Sciences, P.O Box 7072, Kampala, Uganda; 2 Department of Obstetrics and Gynaecology, Walter Sisulu University, Private Bag X1, Mthatha, 5117, South Africa; 3 Department of Reproductive Health, Uganda Christian University, P.O Box 4, Mukono, Uganda

**Keywords:** Risk factors, pre-eclampsia, adverse neonatal outcomes, Mulago hospital, Uganda

## Abstract

**Introduction:**

Pre-eclampsia, which is more prevalent in resource-limited settings, contributes significantly to maternal, fetal and neonatal morbidity and mortality. However, the factors associated with these adverse outcomes are poorly understood in low resource settings. In this paper we examine the risk factors for adverse neonatal outcomes among women with pre-eclampsia at Mulago Hospital in Kampala, Uganda.

**Methods:**

Pre-eclampsia, which is more prevalent in resource-limited settings, contributes significantly to maternal, fetal and neonatal morbidity and mortality. However, the factors associated with these adverse outcomes are poorly understood in low resource settings. In this paper we examine the risk factors for adverse neonatal outcomes among women with pre-eclampsia at Mulago Hospital in Kampala, Uganda.

**Resuls:**

Predictors of adverse neonatal outcomes were: preterm delivery (OR 5.97, 95% CI: 2.97-12.7) and severe pre-eclampsia (OR 5.17, 95% CI: 2.36-11.3).

**Conclusion:**

Predictors of adverse neonatal outcomes among women with pre-eclampsia were preterm delivery and severe pre-eclampsia. Health workers need to identify women at risk, offer them counseling and, refer them if necessary to a hospital where they can be managed successfully. This may in turn reduce the neonatal morbidity and mortality associated with pre-eclampsia.

## Introduction

Pre-eclampsia is a multisystem disorder of pregnancy characterized by hypertension and proteinuria in the second half of pregnancy [1]. It complicates 5-10% of all pregnancies [2, 3], but may be higher in resource-limited settings [4, 5]. Together with other hypertensive diseases of pregnancy, it is one of the leading cause of maternal, fetal and neonatal mortality and morbidity especially in resource limited-settings [4, 6, 7] where diagnosis and obstetric management are deficient [8, 9]. In a study conducted in three hospitals in Uganda, severe pre-eclampsia/eclampsia comprised 8% of cases of severe maternal morbidity [10] and in another study in Mulago Hospital, Uganda, severe pre-eclampsia/eclampsia accounted for 12% of maternal deaths among women with severe maternal morbidity [11].

The perinatal outcomes in women with pre-eclampsia are poor, especially if pre-eclampsia is of early onset and is severe. Haddad and colleagues [12], for example, studied maternal and perinatal outcomes among 239 severe pre-eclamptic women at 24 and 34 weeks of gestation and observed a perinatal mortality of 5.4% although the neonatal morbidities were high especially in early gestation. Similarly, Jantasing and Tanawattnacharoen [7] reported perinatal outcomes in 99 severe pre-eclamptic women between 24 and 34 weeks gestation. The perinatal mortality was 11% and the perinatal morbidity was high especially in lower gestational age mothers.

The etiology of pre-eclampsia is elusive [13] and management depends on early detection, antihypertensive treatment, seizure prophylaxis and rapid delivery in severe cases [1]. Pre-eclampsia is a leading cause of iatrogenic premature delivery [14, 15]. Because pre-eclampsia is a progressive disorder, delivery minimizes severe maternal morbidity although it increases neonatal morbidity and mortality because of prematurity [16]. Neonatal survival depends on gestational age at delivery and is low in small-for-gestational age babies [17].

It is difficult to identify mothers with pre-eclampsia whose neonates will develop adverse outcomes [18]. However, previous studies in high-resource settings have highlighted possible factors that may lead to adverse neonatal outcomes. These include recurrent pre-eclampsia [19, 20], preexisting hypertension [21, 22] and severe hypertension [21–23].

Women with recurrent pre-eclampsia have increased adverse neonatal outcomes because recurrent pre-eclampsia develops early and is severe [19, 20]. Therefore, they are more likely to deliver preterm and small-for-gestation age babies. These infants are prone to have respiratory distress syndrome, have low 5 minute apgar score, and need surfactant, ventilators and admission to the neonatal intensive care unit [21]. Severe hypertension, a feature of severe pre-eclampsia and preexisting hypertension [19, 20], and advanced maternal age [24] are associated with adverse neonatal outcomes because these mothers are prone to abruptio placenta, preterm delivery and neonatal deaths.

Previous studies [21, 25], have examined maternal and neonatal outcomes mainly in high-resource countries where the prevalence of pre-eclampsia is low. The purpose of this study was to assess the maternal characteristics that are associated with adverse neonatal outcomes among women with pre-eclampsia in a resource-limited-setting with a high prevalence of pre-eclampsia.

## Methods

**Design:** A cross-sectional study of pregnant women with pre-eclampsia was carried out from May 2008 to May 2009.

### Setting

The study was conducted in Mulago Hospital's labor wards. Mulago Hospital is Uganda's National Referral Hospital and serves as the teaching hospital for Makerere University College of Health Sciences. Many women with complications of pregnancy, including pre-eclampsia, are referred to Mulago Hospital for management. The hospital carries out approximately 70 deliveries per day and on average six women with pre-eclampsia present to the hospital each day.

### Study population

The study population consisted of 171 women aged 15-39 years who had been diagnosed with pre-eclampsia in Mulago Hospital, Department of Obstetrics and Gynaecology between May 2008 and May 2009. Among women who were enrolled, we identified women who developed adverse neonatal outcomes and compared their socio-demographic characteristics, past medical and obstetric history with those of women who had good outcomes.

Pre-eclampsia was defined according the criteria of the International Society for the Study of Hypertension in Pregnancy [26]. Under this classification, hypertension was defined as a blood pressure of ≥140/90mmHg. The blood pressure was taken with a woman in sitting position using a mercury sphygmomanometer (DT-S101) after 10 minutes of rest. The blood pressure measurement was repeated after four hours. Significant proteinuria was defined as ≥2+ protein by dipstick on two urine samples taken 4 hours or more apart. This was confirmed by a 24hr urine collection of >300mg of protein. Pre-eclampsia was defined as hypertension and significant proteinuria developing after 20 weeks of pregnancy.

Pre-eclampsia was defined as severe if a woman had one or more of the following symptoms: a blood pressure of ≥160mmHg systolic or ≥110mmHg diastolic, ≥ 3+ protein by dipstick on two urine samples taken four hours or more apart or 5g of protein in a 24hr urine sample, epigastric or right upper-quadrant pain, blurring of vision, cerebral disturbances, abnormal liver function, pulmonary edema or cyanosis, fetal growth restriction, low platelets and oliguria of less than 500 ml in 24 hours [1].

### Selection of participants

Pregnant women were eligible for inclusion if they were diagnosed with pre-eclampsia and were at least 20 weeks pregnant. Women were excluded if they had serious medical conditions like pre-existing renal diseases, hypertension, diabetes mellitus, and eclampsia or if they were carrying multiple pregnancies. Women carrying multiple pregnancies were excluded because multiple pregnancies are typically associated with low birth weight (one measure of adverse neonatal outcomes).

Women with pre-eclampsia were selected daily using systematic sampling in which every third mother with pre-eclampsia was selected if she satisfied the inclusion criteria. A sample of 171 women was selected by research assistants who were trained midwives. Sample size was calculated using a formula for cross-sectional studies [27]. We assumed the expected proportion of still births to be 23% as was found in a study by Kharb [28] among Indian women, with 95% confidence interval, power of 80%, with an odds ratio of at least two.

### Explanatory variables

At recruitment, women's socio-demographic characteristics, past medical and obstetric histories were collected through an interviewer administered questionnaire. Blood was drawn at the time of recruitment for complete blood counts, renal and liver function tests. Mid - stream urine samples were taken from the women for random urine protein estimation by dipstick and urine was collected for a 24hr urine protein measurement. All women in this hospital undergo routine counseling and testing for HIV and information about the women's HIV status was collected.

The socio-demographic characteristics included women's age, marital status, education level and socio-economic status. Socioeconomic status was assessed using a household asset index derived using principal components analysis [29]. Measures assessed in the household asset index included type of house (e.g., wall material, floor material and the roofing material), household ownership of assets (e.g., fridge, radio, bicycle, car, motorcycle, and vehicle) and amenities (e.g., water source, electricity and the toilet facility). We also collected data on the women's medical history including previous diagnosis of diabetes mellitus and hypertension, as well as family history of hypertension and renal disease. Obstetric history included information on previous pregnancies and their outcomes and the first day of the last normal menstrual period.

### Outcome measures

Adverse neonatal outcomes were recorded during the women's stay at the hospital. An adverse neonatal outcome included one or more of the following: delivery of a stillborn baby, an early neonatal death, a need to admit the baby to the special care unit, a baby who needed oxygen resuscitation and, a baby weighing less than 2500gm. A still birth was defined as delivery of a baby that died in the uterus after 24 weeks of gestation. An early neonatal death was defined as death of a baby in the first seven days after delivery. Oxygen resuscitation was defined as infants whose partial oxygen pressures were low and who needed oxygen resuscitation. In Mulago Hospital, the period of viability is at least 24 weeks of gestation. Therefore, in this study, delivery of a dead fetus aged less than 24 weeks was not classified as an adverse outcome.

### Statistical analysis

The data collected were coded and entered in Epi Data 3.1 software package. It was transferred to Stata version 10 for analysis. The frequency distributions of the maternal socio-demographic, medical and obstetric characteristics were examined and presented. Bivariate analyses were conducted to assess the association between adverse neonatal outcomes and the maternal socio-demographic, medical and obstetric factors. A p-value less than 0.05 was considered statistically significant.

To control for confounding, we employed multivariable logistic regression analysis. We included all the socio-demographic factors, medical factors and obstetric factors with a p-value of 0.1 or less in the bivariate analysis (e.g, maternal age, parity, systolic blood pressure and diastolic blood pressure) and factors which we thought a priori might be associated with adverse neonatal outcomes (e.g., marital status, educational level, socio economic status, HIV status, and family history of hypertension and plasma vitamin C). Factors with large p-values were eliminated until a stable model was obtained. The results are reported as adjusted odds ratios with their corresponding 95% confidence intervals.

### Ethical consideration

This study was approved by the Makerere University College of Health Sciences Ethics Committee, the Mulago Hospital Ethics Committee and the Uganda National Council for Science and Technology. Written informed consent was obtained from the participants.

## Results

There were 171 women with pre-eclampsia who delivered singleton babies. Most women were married (79.5%), 59.5% had secondary level of education or higher, 50.8% stayed 5km or more from hospital, 1.6% were smokers, 16.3% consumed alcohol and 8.0% were HIV positive.

The neonatal outcomes are shown in Table 1.

**Table 1:** Neonatal outcomes in women with pre-eclampsia

Characteristic	n (%)
**Birth weight**	
<1500	18(10.6)
1500-2400	59(34.9)
≥2500	92(54.4)
**Mode of delivery**	
Normal	74(43.8)
Emergency Caesarean Section	95(56.2)
**Birth Outcome**	
Still births	22(13.1)
Early neonatal deaths	15(8.9)
Live births	1316(77.9)
**Admission to neonatal intensive unit**	
Yes	54(32.1)
No	114(66.9)
**Apgar score**	
0	22(12.9)
1-6	15(8.8)
7-10	133(78.2)

Low birth weight (<2500gm) constituted 45.5%, still birth delivery was 13.1% and early neonatal deaths were 8.9% of the deliveries among women with pre-eclampsia.

Ninety nine out of the 171 women with pre-eclampsia with singleton babies (57.9%) had adverse neonatal outcomes. At the bivariate level, women who were multiparous (p=0.002), women who had severe pre-eclampsia (p=0.001), and women who delivered preterm (p=0.001) were more likely to experience adverse neonatal outcomes (Table 2). After adjusting for other variables, women who had severe pre-eclampsia had 5.2 higher odds of developing adverse neonatal outcomes than women who had mild or moderate pre-eclampsia. Similarly, women who delivered preterm had 5.9 higher odds of developing adverse neonatal outcomes than women who delivered at term (Table 3).

**Table 2:** Background characteristics of women with pre-eclampsia

Characteristic	Adverse neonatal outcomes		Crude Odds Ratio	P Value
	No	Yes		
	n (%)	n (%)		
**Age in years**				
≤24	52(72.2)	56(56.6)	--	-
≥25	20(27.8)	43(43.4)	1.99(1.04-3.82)	0.037
**Marital Status**				
Married	55(76.4)	80(80.8)	-	
Single	17(23.6)	19(19.2)	0.76(0.36-1.61)	0.48
**Educational level**				
Primary or less	34(47.2)	38(38.4)	-	
Secondary or more	38(52.7)	61(61.6)	1.43(0.77-2.65)	0.24
**Socio economic**				
Low	21(29.2)	36(36.7)	-	
Middle income	26(36.1)	29(29.5)	0.65(0.30-1.38)	0.26
High	25(34.7)	33(33.7)	0.77(0.36-1.62)	0.49
**HIV**				
Positive	04(5.6)	08(6.3)	0.65(0.18-2.26)	0.5
Negative	68(94.4)	89(91.8)	-	
**Hypertension in family**				
Yes	30(41.7)	45(58.3)	-	
No	42(58.3)	54(54.6)	0.85(046-1.58)	0.62
**Parity**				
1	51(71.8)	47(47.5)	-	
≥ 2	20(28.2)	52(52.5)	2.82(1.47-5.41)	0.002
**Delivery**				
Normal	35(48.6)	39(39.4)	-	-
Caesarean	37(51.4)	60(60.6)	1.45(0.78-2.68)	0.23
**Pre-eclampsia**				
Mild-Moderate	41(56.9)	24(24.2)	-	
Severe	31(43.1)	75(75.8)	4.13(2.14-7.95)	0.001
**Gestational age**				
≤36 weeks	19(26.4)	66(66.7)	5.57(2.85-10.9)	0.001
≥37 weeks	53(73.6)	33(33.3)		
**Pre-eclampsia history**				
Yes	01(1.4)	10(10.1)	0.12(0.15-1.00)	0.05
No	71(98.6)	89(89.9)		
**Vitamin C**				
<0.11	09(12.5)	12(12.1)	-	
0.11-0.2	47(65.3)	57(57.6)	0.90(0.35-2.34)	0.84
>0.2	16(22.2)	30(30.3)	1.40(0.48-4.04)	0.52
**ANC attendance**				
≤2 times	41(56.9)	60(60.6)	-	0.6
≥3 times	31(43.1)	39(39.40	0.86(0.46-1.59)	
**Smoking**				
Yes	01(1.39)	02(2.0)	-	0.75
No	71(98.6)	97(98.0)	0.68(0.06-7.68)	
**Alcohol**				
Yes	07(9.7)	22(22.2)	-	0.03
No	65(90.3)	77(77.8)	0.37(0.15-0.94)	
**Total**				
**(N=171)**	72(42.1)	99(57.9)		

* ANC antenatal clinic

**Table 3:** Factors independently associated with adverse neonatal outcomes in women with pre-eclampsia: results from logistic regression models

Characteristic	Crude Odds Ratio	Adjusted Odds ratio
**Age in years**	-	
≤24	1.99(1.04-3.82)	Ref
≥25		1.69(0.69-4.1)
**Educational level**		
Primary or less	-	Ref
Secondary or more	1.43(0.77-2.65)	1.5(0.72-3.41)
**Parity**		
1	-	Ref
≥2	2.82(1.47-5.41)	1.71(0.69-4.22)
**Pre-eclampsia**		
Severe	4.13(2.14-7.95)	5.17(2.36-11.3)
Mild-Moderate	-	Ref
**Gestational age**		
≤36 weeks	5.57(2.85-10.9)	5.97(2.79-12.7)
≥37 weeks	-	Ref
**Pre-eclampsia history**		
Yes	0.12(0.15-1.00)	0.12(0.01-1.28)
No	-	Ref

## Discussion

Pre-eclampsia is one of the leading causes of adverse maternal and child outcomes. The risk factors associated with adverse neonatal outcomes in women with pre-eclampsia in resource-limited settings are poorly understood. In this paper we examined the risk factors associated with adverse neonatal outcomes in women with pre-eclampsia.

The risk factors for adverse neonatal outcomes were severe pre-eclampsia and preterm delivery. In this study women with severe pre-eclampsia were at an increased risk of delivering an infant which developed an adverse outcome. This is similar to what was found by other researchers [12, 30, 31]. Buchibinder and colleagues [30], in Ohio in the United States reported that the perinatal mortality in women with severe pre-eclampsia was 8.9% and there was a high perinatal morbidity. Jenkins and colleagues [31] studied maternal and neonatal outcomes in women with severe pre-eclampsia before 25 weeks gestation and observed that only 10% of the neonates survived with major morbidities. Haddad and colleagues [12] studied the maternal and perinatal outcomes during expectant management of women with severe pre-eclampsia between 24 and 33 weeks gestation. The still birth rate was 2.5% and neonatal rate was 3% but with high neonatal morbidity. Perinatal mortality and morbidity were highest in women who developed severe pre-eclampsia at an earlier gestational age and improved with increasing gestational age and where mothers were managed conservatively with prolongation of the pregnancy. Women with severe pre-eclampsia have decreased uteroplacental blood flow and ischemia which compromises blood flow to the fetus [32]. These increases the chances that a mother with severe pre-eclampsia will deliver a baby that will develop an adverse outcome.

In our study, women who delivered preterm were at an increased risk of adverse neonatal outcomes. This is similar to what has been found by other researchers. Khashu and colleagues [33] studied perinatal outcomes associated with preterm birth at 33 to 36 weeks? gestation and found perinatal mortality rate to be 8 times higher, neonatal mortality rate to be 5.5 times higher and, respiratory morbidity to be 4.4 times higher in the pre-term babies than in term babies. Similarly, Young and colleagues [34] studied mortality in late preterm new born babies and found the neonatal mortality rate to be higher in preterm babies than babies born at term.

Pre-eclampsia is a progressive disorder and the only definitive management is the delivery of the fetus [1] to minimize the maternal morbidity and mortality. However, this increases the chance of premature delivery with low odds of child survival [35]. As noted, preterm babies are more likely to be admitted to the neonatal intensive care unit, have assisted ventilation, to be of low birth weight and small for gestational age, and to develop respiratory distress syndrome than term infants [36, 37]. This undoubtedly increases the cost of hospital stay. Given the resource-constraints within the health system, early detection of pre-eclampsia is critical. This can be achieved by ensuring that all maternity facilities are able to provide basic emergency obstetric care. At minimum, maternity facilities should have a functioning blood pressure machine and urine sticks for measuring proteinuria.

Study findings should be interpreted in light of several limitations. In particular, the study sample was relatively small and some predictors had small numbers, therefore the results interpreted with caution as they may not be representative of the general population. In addition, the study was conducted in the national referral hospital and findings may not be generalizable to all women presenting with pre-eclampsia in Uganda. However, this study has enabled us to identify predictors for adverse neonatal outcomes in our setting which can be used to predict and prevent them in resource limited settings.

## Conclusion

In this study, the predictors of adverse neonatal outcomes in pre-eclamptic women were severe pre-eclampsia and preterm delivery. Health workers need to identify mothers at risk, offer them counseling and refer them to a hospital where pre-eclampsia can be managed. This is expected to reduce the perinatal morbidity and mortality associated with pre-eclampsia in resource-limited settings.
